# Assessment of Urinary Dopamine and Serotonin Metabolites in Relation to Dysbiosis Indicators in Patients with Functional Constipation

**DOI:** 10.3390/nu16172981

**Published:** 2024-09-04

**Authors:** Jan Chojnacki, Tomasz Popławski, Aleksandra Kaczka, Natalia Romanowska, Cezary Chojnacki, Anita Gąsiorowska

**Affiliations:** 1 Department of Clinical Nutrition and Gastroenterological Diagnostics, Medical University of Lodz, 90-647 Lodz, Poland; aleksandra.kaczka@umed.lodz.pl (A.K.); cezary.chojnacki@umed.lodz.pl (C.C.); 2 Department of Pharmaceutical Microbiology and Biochemistry, Medical University of Lodz, 92-215 Lodz, Poland; tomasz.poplawski@umed.lodz.pl; 3 Department of Gastroenterology, Medical University of Lodz, 92-213 Lodz, Poland; natalia.romanowska1@stud.umed.lodz.pl (N.R.); anita.gasiorowska@umed.lodz.pl (A.G.)

**Keywords:** functional constipation, dopamine, homovanillic acid, serotonin, 5-hydroxyindoleacetic acid, gut microbiome, dysbiosis index

## Abstract

Background: The causes of functional constipation (FC) in adults are unclear, but changes in the gut microbiome may play an important role. The present study aimed to assess the relationship between urinary metabolites of dopamine and serotonin and some dysbiosis indicators in patients with FC. The study included 40 healthy women and 40 women with FC aged 21–46 years. Methods: Urinary levels of homovanillic acid (HVA), 5-hydoxyindoleacetic acid (5-HIAA), p-hydroxyphenylacetic acid (PhAc), and 3-indoxyl sulfate, as final metabolites of dopamine, serotonin, and indole pathway, respectively, were determined using the LC-Ms/Ms method. However, hydrogen–methane and ammonia breath tests were performed. The GA-map Dysbiosis Test was used to identify and characterize the dysbiosis index (DI). Results: In patients with FC, the DI was significantly higher than in the control group: 4.05 ± 0.53 vs. 1.52 ± 0.81 points (*p* < 0.001), but the number of many types of bacteria varied among individuals. The levels of HVA were higher, while 5-HIAA levels were lower in patients. Moreover, the HVA/5-HIAA ratio had a positive correlation with DI as well as with the severity of symptoms. Conclusions: In patients with functional constipation, the balance in dopamine and serotonin secretion is disturbed, which is associated with changes in the gut microbiome.

## 1. Introduction

Functional disorders of the gastrointestinal tract are still a complex problem in clinical practice. This is due to the complexity of their pathogenesis [1]. Genetic predisposition is considered among the pathogenic factors, but environmental factors are also important. The diagnosis is based mainly on the symptoms reported by the patient, including their nature and severity. This information was also used to develop diagnostic criteria. The Rome IV Criteria distinguish 21 functional diseases in adults [2,3]. The most common one is irritable bowel syndrome (IBS), functional constipation (FC), and functional diarrhea [FD]. The clinical picture of IBS is varied. There is a type with predominant constipation (IBS-C), diarrhea (IBS-D), a mixed type (IBS-M), and an unspecified type (IBS-U). Common symptoms are abdominal pain and bowel movement disorders. These ailments occur chronically with a tendency to convert [4,5,6]. Additionally, the nature of symptoms may change over time. In many patients, the IBS subtype changes over several months or years [7,8]. Unlike IBS, the symptoms of FC are relatively constant, and abdominal pain is not predominant and not related to the act of defecation [9,10]. Research on symptom variability has examined the role of nutritional factors, including amino acids such as phenylalanine and tryptophan, which are substrates for producing dopamine and serotonin, respectively [11,12]. Both neurotransmitters are synthesized in the central and visceral nervous systems and the digestive tract cells [13,14]. They play an essential role in regulating their functions. Serotonin, with the participation of numerous receptors, mainly stimulates intestinal motor activity [15,16]. Many researchers have shown increased serotonin levels in IBS-D patients [17,18,19]. Others did not confirm such a simple relationship between serotonin levels and the nature of bowel symptoms [20,21]. These studies did not consider other factors, such as dopamine, which is also secreted in large amounts by different cells of the gastrointestinal tract (GIT) [22,23,24]. Unlike serotonin, it relaxes the GIT and plays an essential role in the pathogenesis of functional disorders of the digestive tract [25,26,27]. Disturbances in the homeostasis of serotonin and dopamine may be one of the causes of functional diseases of the GIT. The balance between these neurotransmitters can be disturbed by many factors, including changes in the gut microbiome [28,29]. In addition, many bacterial strains can synthesize serotonin as well as dopamine [30,31,32]. In our previous study, we found significant differences in the serotonin and dopamine levels in patients with IBS-C and IBS-D [33].

The current study aimed to assess the urinary level of the homovanillic acid 5-hydroxyindoleacetic acid and selected indicators of dysbiosis in patients with functional constipation.

## 2. Materials and Methods

### 2.1. Participants

The study included 40 women without any abdominal complaints (Group I, Controls) and 40 women with functional constipation (Group II), aged 29–46 years. All participants were recruited and under the care of a gastroenterology clinic from September 2019 to March 2023. Criteria for including women in the control group: age below 50 years, regular monthly cycles, typical laboratory test results, exclusion of any diseases except tension headaches (4 people), periodic chronic fatigue (3), and sleep disorders (11)

The patients’ inclusion criteria are consistent with the Rome IV Criteria, which are fulfilled for the last three months, two or more of the following symptoms above 25% of the time: straining, lumpy or hard stools, sensation of anorectal obstruction, manual evacuation, and fewer than three spontaneous bowel movements per week. The duration of the disease was from 4 to 11 years. The bothersome symptoms persisted despite taking various pharmacological agents.

Exclusion criteria for both groups: inflammatory bowel diseases, celiac disease, allergy, liver and pancreas disease, thyroid diseases, diabetes and other metabolic diseases, mental disorders, the use of antibiotics, probiotics, hormones, and psychotropic drugs.

A constipation scoring system was used to assess the severity of constipation [34]. The following symptoms were included: frequency of bowel movements, difficult or painful evacuation, completeness of evacuation, abdominal pain, time per attempt, type of help (laxatives, enema), number of unsuccessful attempts at evacuation in 24 h, and duration of constipation. The total score range was from 0 to 30.

### 2.2. Dysbiosis Test

The GA-map Dysbiosis Test (GA-map Dysbiosis Test, Genetic Analysis, Oslo, Norway) [35] was technically documented following EU requirements and was used to identify and characterize dysbiosis by differences between the study groups. This test allows the mapping of the intestinal microbiota profile for a selected set of bacteria. This method uses 54 bacterial markers based on the 16S rRNA sequences in seven variable regions (V3–V9) and measures the relative abundance of bacteria. Each bacterial DNA marker is a source of different bacterial species or groups, which allows the detection of over 300 bacteria from various taxonomic categories. In our study, the following species of bacteria were tested: *Actinobacteria*, *Bacteroidetes*, *Firmicutes*, *Proteobacteria*, *Tenericutes*, and *Verrucomicrobia* (FloraGen test (polish name for GA-map Dysbiosis Test), ALAB Laboratories, Warsaw, Poland), which are the most common in the Polish population. A unique algorithm described in [35] facilitates the determination of dysbiosis level and deviation from a normobiotic state in a clinical setting. Briefly, the dysbiosis index (DI) is calculated using the specialized GA-map^®^ Analyzer Software v1.4, provided by the kit vendor from 1 to 5, where a value of 2 or lower is no-dysbiotic, and a higher DI above 2 confirms dysbiosis. The value of the DI was clinically standardized and validated on the European healthy population. This index refers to quantitative changes and proportions between intestinal bacteria.

### 2.3. Laboratory Tests

The laboratory tests were performed, including blood cell count, glucose, glycated hemoglobin, profile of lipids, bilirubin, iron, urea, creatinine, thyroid stimulating hormone, free thyroxine and triiodothyronine, antibodies to tissue transglutaminase, liver and pancreas function indicators, *C*-reactive protein, deaminated gliadin peptide, fecal calprotectin, and metabolites of assessed neurotransmitters in urine. Urine samples for phenylalanine and tryptophan metabolites testing were collected in the morning into a special container with a 0.1% hydrochloric acid solution as a stabilizer. We determined the concentration of the following metabolites in urine: homovanillic acid (HVA), 5-hydroxyindoleacetic acid (5-HIAA), -p-hydroxyphenylacetic acid (PhAc), and 3-indoxyl sulfate (Indican, 3-IS)). These metabolites were determined in the programs Organix Neuro and Organix Gastro (ALAB laboratories, Warsaw, Poland), using liquid chromatography with tandem mass spectrometry (LC-MS/MS, (Ganzimmun Diagnostics AG, Mainz, Germany). The above metabolites were treated as indicators of the dopamine, serotonin, and indole pathways, respectively.

### 2.4. Breathing Test

The hydrogen–methane breath test (HBT) was performed using a Gastrolyzer (Bedfont, Ltd., Harrietsham, UK). The levels of hydrogen and methane in breath were measured fasting and after ingestion of 10 g of lactulose by patients, dissolved in 200 mL of water. Breath samples were collected at 15 min intervals for 3 h. The criterion for a positive SIBO diagnosis was a minimum increase of 20 ppm of hydrogen and 10 ppm of methane within the first 90 min of testing.

The ammonia breath test (ABH) was performed using a gas analyzer (HELIC ABT Reader, AMA Co., Ltd., Mikkeli, Finland). The ammonia concentration (NH3) in the expiratory air was determined by fasting and then at 15-min intervals for 3 h after ingesting 250 mL of protein solution (Nutridrink Protein—Nutricia).

After discontinuing antibiotics, probiotics, and drug-inhibiting gastric secretion, both tests were performed for four weeks. The concentration of the above ions (hydrogen, methane, ammonia) at 0, 90, and 150 min of the tests was assumed for statistical analysis.

### 2.5. Nutritional Intervention

All participants were recommended to maintain their current diet but with limited phenylalanine (PhA) and tryptophan (TRP) intake to 40 mg and 15 mg per kilogram of body weight, respectively, for 30 days before investigations. The average of their consumption was calculated using the nutritional calculator with the Kcalmar—pro-Premium application (https://kcalmar.com/dietetyk/o-aplikacji/, accessed on 31 August 2024; Hermex, Lublin, Poland). The patients applied the balanced diet with a total caloric value of 2000 kcal and a daily intake of 50 g of protein, 270 g of carbohydrates, 70 g of fats, and 30 g of dietary fiber. Everyone was administered a diet the day before the examination, with the PhA and TRP content calculated earlier. The use of any drugs was forbidden except laxatives. Patients from both groups were recommended to complete a diet diary daily, under the control of competent dietitians, with whom they had telephone and e-mail contact. The research was conducted as an open-label clinical trial.

### 2.6. Ethical Issues

The study was conducted according to the guidelines of the Declaration of Helsinki and the Guidelines for Good Clinical Practice and approved by the Bioethics Committee of the Medical University of Lodz (RNN/176/18/KE).

### 2.7. Data Analysis

We first checked the normality of data distribution using the Shapiro–Wilk W test. We used the Student’s *t*-test and the Wilcoxon matched-pairs signed rank test to compare two paired groups. For two unpaired groups, we used the Mann–Whitney test. Simple linear regression was conducted using the Spearman correlation with the rho rank correlation coefficient (t). The Wilcoxon signed-rank test was used to analyze group differences before and after treatment. We employed the Two-Proportions Z-Test to compare the occurrence of various bacteria in the study group. Correlations were analyzed using the Spearman rank test with the rho rank correlation coefficient (r). We considered differences significant at *p* < 0.05. The effect size (θ) was calculated as reported elsewhere. All statistical analyses were performed using STATISTICA 13.3 software (TIBCO Software Inc., Palo Alto, CA, USA).

## 3. Results

There were no differences in the results of routine laboratory tests between the two study groups, except for fecal calprotectin. However, the results in both groups did not exceed the reference values (Table 1).

The dysbiosis index (DI) in the control group was 1.52 ± 0.81 points, and in the patients with functional constipation it was significantly higher—4.05 ± 0.53 points (*p* < 0.001; θ = 0.861). In healthy people, the dispersion of the results of DI was quite large but did not exceed negative and positive reference points. Reference points 2 and 3 were taken as bacterial reduction or increase exponent. In the group of patients, both a decrease and an increase in the number of various species of bacteria were observed. In particular, more patients had an increase in bacteria compared to the number of patients with a reduction: *Bifidobacterium* spp. (*p* < 0.001), *Bacteroides fragilis* (*p* < 0.001), and *Clostridium* spp. (*p* < 0.01). A smaller number of patients showed a reduction in bacteria, especially in *Lactobacillus* spp. (*p* < 0.01) and *Ruminococcus* (*p* < 0.05, Table 2). Changes in other species of bacteria were insignificant.

In the study group, a higher concentration of hydrogen ions was found on an empty stomach and in the 90th minute of the lactulose breath test. In the same test, higher methane concentrations in patients were found at 90 and 150 min. No differences in ammonia concentrations were found in any time interval (Table 3).

**Figure 1:**
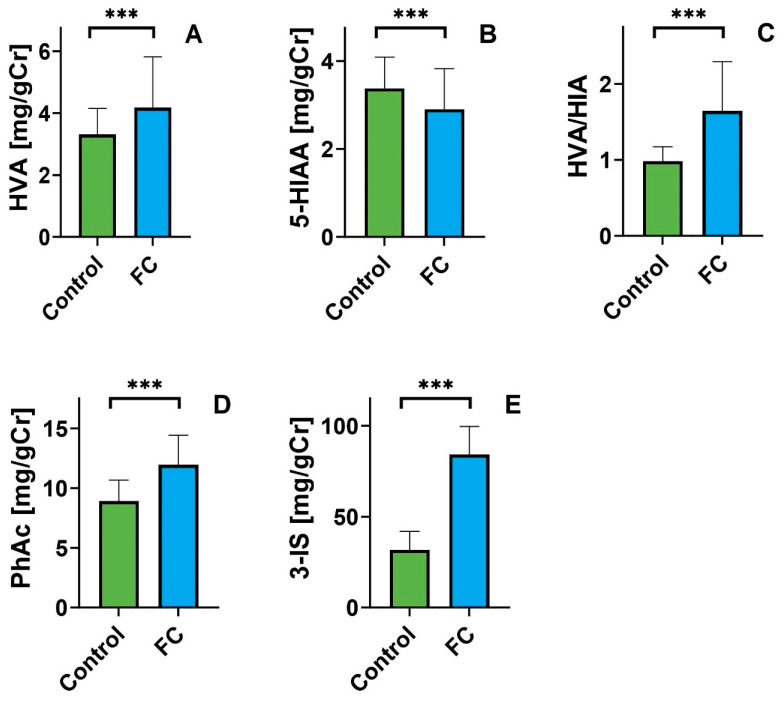
Urinary levels of hydroxyvanillic acid ((**A**); HVA), 5-hydroxyindoleacetic acid ((**B**); 5-HIAA), hydroxyvanilic acid/5-hydroxyindoleacetic acid ratio ((**C**); HVA/HIIA),p- hydroxyphenyl acetic acid ((**D**); PhAc) and 3-indoxyl sulfate ((**E**); 3-IS) expressed in milligrams per gram of creatinine (mg/gCr) in the control group (green) and patients with functional constipation (blue). Data are presented as Mean; error bars represent SD values. Differences between controls and patients were evaluated by the Student’s or the Mann–Whitney U test; *** *p* < 0.001; average ± SD; Student’s *t*-test assessed differences between groups.

Compared to the control group, patients with constipation had a statistically higher level of homovanillic acid (4.17 ± 1.64 vs. 3.30 ± 0.84 mg/gCr; θ = 0.272) and lower levels of hydroxyindole acetic acid (2.90 ± 0.91 vs. 3.37 ± 0.71 mg/gCr; θ = 0.230). Similar differences occurred in the HVA/5-HIAA ratio (θ = 0.638) (Figure 1). The differences in dopamine metabolite (HVA) levels between FC patients and controls suggest increased dopamine production in the patient group. We also observed that FC patients produce significantly less serotonin than the control group, indicating an inverse relationship for serotonin metabolites (5-HIAA). These changes are likely due to differences in tryptophan bioavailability in the study groups. The difference in the levels of metabolites of the indole metabolism pathway (PhAc, and 3-IS) evidences this. The higher the level, the more the balance of tryptophan metabolism is shifted toward its degradation, resulting in reduced bioavailability for serotonin production. The levels of metabolites of the indole pathway, PhAc, and 3-IS, were higher in the patient’s group (θ = 0.564 and θ = 0.858, respectively; Figure 1).

**Figure 2:**
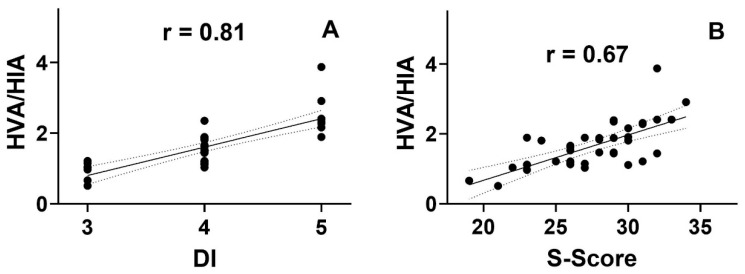
Correlation between the dysbiosis index ((**A**); DI) and the severity of symptoms ((**B**); S-score) and the urinary levels of homophilic acid (HVA) and 5-hydroxy acetic acid (5-HIAA) ratio (HVA/HIA) in patients with functional constipation (n = 40). The correlations were analyzed using the Spearman rank test with the rho rank correlation coefficient (r).

The dysbiosis index positively correlated with the severity of clinical symptoms (Spearman rank test; 0.68; *p* < 0.05). There was a similar relationship between the HVA/5-HIAA ratio and between DI (Spearman rank test; 0.81 and; *p* > 0.05) and S-score (Spearman rank test; 0.67; *p* > 0.05) (Table 4, Figure 2). No other statistically significant correlations were observed. However, there is a negative tendency between the values of 5-HIAA and HVA and between HVA and 3-IS.

## 4. Discussion

The results indicated that patients with FC significantly differ in urine levels of HVA and 5-HIAA compared to healthy people. In both groups, the intake of their substrate, i.e., phenylalanine and tryptophan, was maintained the same during the study. Other factors are responsible for changes in their metabolism. Decreased 5-HIAA concentration in urine has previously been found in postmenopausal women suffering from constipation [36]. In these patients, no relationship was found between hormonal changes and the severity of symptoms. However, similar changes in serotonin homeostasis were previously found in younger IBS-C patients [33]. The results indicate that neurotransmitters are involved in the development of this syndrome. The gastrointestinal tract (GIT) is the main location in the body for producing serotonin (about 90%) and dopamine (about 50%). Serotonin and dopamine receptors are found throughout the digestive tract. The secretion of these neurotransmitters and the expression of their receptors can change due to various factors, including intestinal bacteria, as suggested by the results presented. In the current study, we also found an increase in the metabolites of the indole pathway, particularly in the level of 3-indole sulfate. This metabolite is synthesized via tryptophanase of colon anaerobic bacteria and is considered a quantitative biomarker of the intestinal microbiome [37,38]. In turn, p-phenylacetic acid is also produced in the indole pathway, and its source is phenylalanine and tyrosine [11]. High levels of these metabolites may support increased anaerobic bacteria in the intestines. Other indirect indicators also indicate an increased number of bacteria, including the results of breathing tests. Small intestinal bacterial overgrowth (SIBO) usually causes diarrhea, more likely constipation [39]. Clinical interpretation of this variety is not straightforward, mainly when similar lactulose breath test results are obtained in patients with dominant diarrhea and constipation. The value of this test is sometimes questionable [40]. Despite severe diarrhea, the increased breath hydrogen concentration in the initial 90 min after lactulose intake is slight (flat line). This may indicate an overgrowth of bacteria in the distal part of the small intestine and the colon. This justified extending the study to further time intervals [40,41]. In addition, it is estimated that about 15–20% of people with positive hydrogen breath tests do not have any abdominal complaints. A more established opinion is that bacterial overproduction of methane in the intestine is the cause of chronic constipation, but it also occurs in healthy patients. Our experience also supports the need to critically evaluate the results of the hydrogen–methane test with the symptoms of functional intestinal diseases. There is less Ie with ammonIa tests in these diseases. Ihis test is commonly used to diagnose *Helicobacter pylori* infection [42]. Urea is the primary nitrogenous metabolite of protein breakdown. The intensity of this process depends on protein intake and the amount of type of gut bacteria. This process occurs via bacterial hydrolysis of urea by bacterial urease, bacterial protein deamination, and intestinal mucosal glutamine catabolism [43]. Bacteria capable of producing urease include *Klebsiella oxytoca*, *Proteus* spp. *Pseudomonas aeruginosa*, *Yersinia enterocolica,* and others [44]. Ammonia may increase the activity of tryptophan hydroxylase and acetylcholinesterase and consequently increase the synthesis of serotonin and acetylcholine [45]. In our material, the concentration of breath ammonia was similar in both study groups.

Similarly, many other strains of bacteria can directly or indirectly increase the production of neurotransmitters in the gastrointestinal tract, including serotonin and dopamine. The properties of serotonin synthesis are attributed, among others, *to Escherichia coli* [46], *Lactobacillus plantarum*, *Streptococcus hemophilus* [47], and *Staphylococcus* [48]. In turn, dopamine can be synthesized by some strains of *Escherichia coli* [46,48], *Bacillus* spp. [47], *Proteus vulgaris* and *Streptococcus aureus* [49], *Lactobacillus plantarum* [50], and *Enterococcus faecium* [51]. The above findings mainly concern the results obtained in vitro cultures.

Microbiome studies in patients with chronic constipation revealed an increase in many bacteria, such as *Bifidobacterium*, *Bacteroides*, *Clostridia*, *Faecalibacterium*, *Lactobacillus*, and *Methanogens* [52,53,54,55,56]. On the contrary, other researchers in this group of patients found fewer bacteria, including *Bacteroides*, *Bifidobacterium*, *Clostridium*, and *Lactobacillus* [57,58,59].

Changes in the gut microbiome were also found in our patients with FC. However, a high index of dysbiosis was demonstrated in this study group. Other researchers have also demonstrated the usefulness of the GA-map Dysbiosis Test, but it was performed mainly in IBS patients [35,60,61]. There are no studies using this method in patients with FC in the available literature. Nevertheless, in patients with IBS-C, a lower number of protective bacteria were found [62,63].

A detailed microbiome assessment revealed an increase in *Bifidobacterium* spp. and *Bacteroides fragilis*. Both bacteria belong to the anti-inflammatory group and produce short-chain fatty acids [64,65]. However, a reduction in bacteria of the genus *Lactobacillus* spp. was found. These bacteria also have anti-inflammatory properties and reduce the production of serotonin [46] while increasing dopamine production [49]. These changes may affect the clinical picture of functional gastrointestinal diseases. The obtained results do not authorize referring these changes to individual bacteria because all patients showed an increase or decrease in selected bacteria. Simply quantifying taxa has many limitations. The secretory and metabolic properties of bacterial strains in vitro culture requirements.

Numerous studies of IBS patients have found a similar diversity of microbiome changes. A systemic review evaluating 24 studies in IBS patients showed a significant increase in *Enterobacteriaceae* and *Bacteroides* and a decrease in *Bifidobacterium* and *Faecalibacterium* [66]. Other reviews with meta-analysis in patients with IBS found reductions in *Bifidobacterium*, *Lactobacillus*, and *Faecalibacterium* [67] and an increase in *Firmicutes* and *Clostridiales* but a decrease in *Bacteroidales* [68]. Similarly, another meta-analysis in IBS patients found lower *Lactobacillus* and *Bifidobacterium* but higher *Escherichia coli* end *Enterobacter* [69]. In a recent review on the main microbiome alterations, an increase in *Firmicutes* and *Proteobacteria* species was considered, as well as a decrease in *Bacteroides* and *Actinobacteria* phylum in IBS patients [70].

The differences in bacterial diversity between patients from different regions of the word may result from several factors, including different nutrition and eating habits. They also result from the different methods used to study the gut microbiome. Our process is based on DNA bacterial profiling technology, allowing accurate microbiome profile identification. The results of the FloraGen test refer to a reference group of healthy people from the European population. However, our study used a control group, and the results confirmed its validity because the microbiome profile was similar to the accepted standards. The analysis results include 48 selected bacterial markers. There are still few works using this method, and they concern IBS patients.

It is worth emphasizing that the stability of the microbiome is also important. It changes over time for many reasons, including nutritional factors. Changes in the microbiome profile may also occur in healthy people. For this reason, the term dysbiosis has been assigned to changes in the microbiome resulting in clinical symptoms, the “dysbiosis syndrome”. It is also possible for some symptoms to occur with a slight disturbance in the number proportions of intestine bacteria. Changes in the activity of metabolic pathways may cause these symptoms. Our study’s results support distinguishing between profile “dysbiosis” and “metabolic dysbiosis” [71]. The genetic and biochemical indicators of dysbiosis only partially correlate with each other. Nevertheless, these results are complementary and may be helpful when choosing a treatment method. Assessing gut microbiome profiles can help choose the right probiotic, but it is likely when the results are diverse. It is expected that the gut bacteria, through increased metabolism of TRP via the indole pathway, reduce its amount for the production of serotonin [72,73]. Changing these proportions can be achieved by adjusting the intake of TRP and PhAc. The results of genetic and metabolic tests allow for a more accurate determination of the dysbiosis state, which has practical value and facilitates personalized therapy.

Our study has some limitations. The respondents’ groups were insignificant and included only women, in whom FC occurs more often than men. Nutrition assessment depends only on patients’ declarations contained in the diary. The FloraGen method used also has its limitations. Some bacterial markers are specific to one species, but others cover a larger group of bacteria. For example, the genera *Shigella* and *Escherichia* are closely genetically related and therefore difficult to distinguish using methods based on 165 rRNA sequences.

In summary, our results confirm the opinions of other researchers, indicating the vital role of alterations in the gut microbiome in the pathogenesis of functional diseases of GIT. These changes can lead to an imbalance in dopamine and serotonin production. This knowledge justifies using selected dopaminergic and serotoninergic receptors modulating drugs in the symptomatic treatment of this syndrome and calls for further research on the gut microbiome. The results of indirect breath tests (hydrogen, methane, hydrogen sulfide, ammonia) should be an indication for testing using DNA profiling technology. These methods allow for an accurate assessment of the microbiome profile and the implementation of causal nutritional and microbial therapy. Restoration of eubiosis should be the target of further research.

## 5. Conclusions

In patients with functional constipation, the balance in dopamine and serotonin secretion is disturbed, which is associated with changes in the gut microbiome. Treating this disease should aim to achieve intestinal eubiosis.

## Figures and Tables

**Table 1:** Participants’ general characteristics and the selected biochemical parameters in healthy subjects (Group I, Controls) and patients with functional constipation (Group II).

Feature	Group I (n = 40)	Group II (n = 40)	*p*-Value
Age (years)	37.7 ± 5.8	38.0 ± 5.3	0.916
Gender—F	40	40	n/a
BMI (kg/m^2^)	23.1 ± 0.8	23.4 ± 0.5	0.054
GFR (mL/min)	99.3 ± 3.4	97.7 ± 4.3	0.062
ALT (µ/L)	16.6 ± 3.8	15.3 ± 3.9	0.057
AST (µ/L)	14.7 ± 1.9	16.4 ± 1.4	0.092
CRP (mg/L)	3.0 ± 0.8	3.1 ± 0.7	0.192
FC (µg/g)	28.3 ± 4.7	34.6 ± 7.5	0.0007

BMI—body mass index, GFR—glomerular filtration rate, ALT—alanine aminotransferase, AST—aspartate aminotransferase, CRP—*C*-reactive protein, FC—fecal calprotectin; data are presented as average ± SD; differences between groups were assessed by Student’s *t*-test; n/a not applicable

**Table 2:** Number and percentage (No/%) of patients with functional constipation (n = 40) who had a decrease or increase in selected phyla and species of intestinal bacteria as 2 and 3 reference points. Differences in the occurrence of the bacteria were assessed using the Two-Proportions Z-Test.

Bacteria	Decrease	Increase	*p*-Value	95% CI
*Actinomycetales*	3 (7.5)	8 (20.0)	0.052	n/a
*Bifidobacterium* spp.	9 (12.5)	23 (57.5)	0.00069	0.241–0.659
*Bacteroides fragilis*	3 (7.5)	21 (52.5)	0.00006	0.295–0.703
*Clostridium* spp.	6 (15.0)	17 (42.5)	0.003	0.077–0.473
*Faecalibacterium praise*	6 (15.0)	6 (15.0)	0.50	n/a
*Furmicutes* (varia)	9 (22.5)	7 (17.5)	0.28	n/a
*Lactobacillus* spp.	12 (30.0)	4 (10.0)	0.013	n/a
*Prevotella* spp.	4 (10.0)	6 (15.0)	0.25	n/a
*Ruminococcus* (varia)	10 (25.0)	4 (10.0)	0.039	n/a
*Streptococcus* spp.	9 (22.5)	6 (15.0)	0.19	n/a

n/a not available.

**Table 3:** Concentrations of hydrogen, methane, and ammonia in exhaled air in different time intervals of the hydrogen–methane and ammonia tests in healthy people (Group I, n = 40) and in patients with functional constipation (Group II, n = 40); average ± SD, differences between groups were assessed by the Student’s *t*-test.

Ions (Time, min)	Group I (ppm)	Group II (ppm)	*p*-Value	Θ
Hydrogen (0)	6.75 ± 2.1	14.77 ± 5.7	<0.001	0.75
Hydrogen (90)	23.07 ± 5.7	29.80 ± 9.7	<0.001	0.35
Hydrogen (150)	93.45 ± 19.9	99.15 ± 14.9	0.18	n/a
Methane (0)	4.72 ± 1.6	4.72 ± 1.3	0.94	n/a
Methane (90)	4.57 ± 1.1	5.25 ± 1.2	0.01	0.28
Methane (150)	12.07 ± 4.1	15.40 ± 5.7	0.007	0.3
Ammonia(0)	4.12 ± 1.0	4.26 ± 1.0	0.67	n/a
Ammonia (90)	5.37 ± 0.9	5.27 ± 1.0	0.52	n/a
Ammonia (150)	8.92 ± 2.0	8.94 ± 1.7	0.96	n/a

n/a not available.

**Table 4:** Correlation between the dysbiosis index (DI) and the severity of symptoms (S-score) and the level of tested metabolites in urine: HVA—hydroxyphenylacetic acid; 5-HIAA—5-hydroxyindoleacetic acid; PhAc—p-hydroxyphenylacetic acid; 3-IS—3-indoxyl sulfate and HVA/HIA ratio in patients with functional constipation. The correlations were analyzed using the Spearman rank test; significant values (alpha = 0.05) are marked *.

Variable	DI	S-Score	HVA	5-HIAA	HVA/HIAA	PhAc	3-IS
DI	1	0.68 0.00018 *	0.17 (0.31)	−0.12 (0.44)	0.81 0.00019 *	−0.13 (0.42)	0.18 (0.27)
S-Score	0.68 0.00018 *	1	0.20 (0.2)	0.13 (0.93)	0.67 0.00022 *	−0.15 (0.35)	0.11 (0.51)
HVA	0.17 (0.31)	0.20 (0.2)	1	−0.21 (0.19)	0.33 0.034 *	0.01 (0.97)	−0.16 (0.33)
5-HIAA	−0.12 (0.44)	0.13 (0.93)	−0.20 (0.19)	1	−0.11 (0.5)	0.31 (0.052)	−0.69 (0.24)
HVA/HIAA	0.81 0.00019 *	0.67 0.00022 *	0.33 0.034 *	−0.11 (0.5)	1	−0.08 (0.62)	0.23 (0.14)
PhAc	−0.13 (0.42)	−0.15 (0.35)	0.01 (0.97)	0.31 (0.051)	−0.08 (0.62)	1	−0.09 (0.59)
3-IS	0.18 (0.28)	0.11 (0.51)	−0.16 (0.33)	−0.19 (0.24)	0.23 (0.14)	−0.09 (0.59)	1

## Data Availability

The data presented in this study are available.
